# Covalently Binding Adenosine A_3_ Receptor Agonist ICBM Irreversibly Reduces Voltage-Gated Ca^2+^ Currents in Dorsal Root Ganglion Neurons

**DOI:** 10.1007/s11302-023-09929-y

**Published:** 2023-03-15

**Authors:** Federica Cherchi, Martina Venturini, Giada Magni, Mirko Scortichini, Kenneth A. Jacobson, Anna Maria Pugliese, Elisabetta Coppi

**Affiliations:** 1 Department of Neuroscience, Drug Research and Child Health, University of Florence, Viale Gaetano Pieraccini 6, 50139 PsychologyFlorence, Italy; 2 Istituto Di Fisica Applicata “Nello Carrara,” Consiglio Nazionale Delle Ricerche, Via Madonna del Piano 10, 50019 Sesto Fiorentino, Florence, Italy; 3 National Institute of Diabetes and Digestive and Kidney Diseases, National Institutes of Health, Bethesda, MD 20892 USA

**Keywords:** Adenosine receptors, Ca^2+^ currents, Covalent binding, Irreversible agonist, Analgesia, Dorsal root ganglion neurons

## Abstract

Interest has been focused in recent years on the analgesic effects exerted by adenosine and its receptors, A_1_, A_2A_, A_2B_, and A_3_ adenosine receptor (AR) subtypes, in different in vivo models of chronic pain. In particular, it was demonstrated that selective A_3_AR agonists reduced pro-nociceptive N-type Ca^2+^ channels in dorsal root ganglion (DRG) neurons isolated from rats and, by this mechanism, inhibit post inflammatory visceral hypersensitivity. In the present study, we investigate the effect of a previously reported irreversibly binding A_3_AR agonist, ICBM, on Ca^2+^ currents (I_Ca_) in rat DRG neurons. Present data demonstrate that ICBM, an isothiocyanate derivative designed for covalent binding to the receptor, concentration-dependently inhibits I_Ca_. This effect is irreversible, since it persists after drug removal, differently from the prototypical A_3_AR agonist, Cl-IB-MECA. ICBM pre-exposure inhibits the effect of a subsequent Cl-IB-MECA application. Thus, covalent A_3_AR agonists such as ICBM may represent an innovative, beneficial, and longer-lasting strategy to achieve efficacious chronic pain control versus commonly used, reversible, A_3_AR agonists. However, the possible limitations of this drug and other covalent drugs may be, for example, a characteristic adverse effect profile, suggesting that more pre-clinical studies are needed.

## Introduction

Current therapies for the management of pain are inadequate, and new approaches are needed to mitigate its immense societal burden [1]. Opioids, anticonvulsants, nonsteroidal anti-inflammatory drugs (NSAIDs), channel blockers, and antidepressants are widely used, but their clinical efficacy is variable and often with serious adverse effects [1–3].

One of the promising directions for future pain therapy is the neuromodulator adenosine, which acts through G protein-coupled receptors (GPCRs). Four adenosine receptors (ARs), comprising the A_1_ and A_3_ subtypes coupled to G_i_ protein and the A_2A_ and A_2B_ subtypes coupled to G_s_ protein [4] has been implicated in pain modulation. Selective A_1_AR and A_3_AR agonists counteract pain behaviors in various acute and chronic models, reviewed by [5–7], while the role of A_2A_AR and A_2B_AR is less well established [8–10]. Furthermore, therapeutic use of A_1_AR- or A_2A_AR-selective agonists is impeded by cardiovascular side effects, resulting from the stimulation of bradycardiac A_1_ receptors in the heart and vasodilatory A_2A_ receptors [11]. However, A_3_AR agonists that are already in phase 2 and 3 clinical trials, e.g., IB-MECA (1-deoxy-1-[6-[[(3-iodophenyl)methyl]amino]-9H-purine-9-yl]-*N*-methyl-β-D-ribofuranuronamide) and its 2-chloro derivative Cl-IB-MECA (2-chloro-*N*^6^-(3-iodobenzyl)-adenosine-5′-*N*-methyluronamide), do not induce cardiac or hemodynamic effects, at least at moderate doses [12]. They display no serious adverse effects in clinical trials for autoimmune inflammatory diseases, liver cancer, and non-alcoholic steatohepatitis in > 1500 human subjects [13], thus suggesting the feasibility of using A_3_AR agonists clinically for other conditions, such as pain. Activation of the A_3_AR was efficacious in reducing pain in multiple mouse and rat models [8, 14–20]. A range of A_3_AR agonists were used in these in vivo studies, including innovative, highly selective (N)-methanocarba adenosine derivatives MRS5841 [21], MRS7220 [22], and MRS7154 [23]; for a review, see [24], among which are the versatile pharmacological probes MRS5980 [25, 26] and MRS5698 [27, 28] and the water-soluble prodrug MRS7476 [29]. Multiple mechanisms of action (MoA) have been proposed to mediate the A_3_AR-induced pain relief, including modulation of spinal neuro-glial communication, neuroinflammation, and the GABAergic system in the spinal cord [30, 31]. We described an additional activity of A_3_AR agonists in 2019, namely decreased firing of isolated nociceptive dorsal root ganglia (DRG) neurons and inhibition of pro-nociceptive Ca^2+^ currents (I_Ca_; sensitive to the N-type Ca^2+^ channel blocker PD173212, an analogue of ω-conotoxin) [32]. Both Cl-IB-MECA and the more selective (> 3000-fold) A_3_AR agonist MRS5980 produced these effects.

Voltage-dependent Ca^2+^ channels (VDCCs) are inhibited by gabapentinoids, a first-line treatment for chronic neuropathic pain, by their blocking Ca_v_2.2 channels upon binding to the α2δ subunits [33, 34]. Presynaptic VDCC inhibition in the peripheral and central nervous system attenuates nociceptive neurotransmitter release. A selective N-type VDCC inhibitor, ω-conotoxin GVIA (ω-CTX), greatly reduces (by 60%) excitatory postsynaptic currents evoked by stimulating lamina I dorsal horn neurons [35], indicating that VDCCs play a key role in regulating nociceptive neurotransmitter release. Moreover, aberrant expression and/or activation of N-type VDCCs correlates with neuropathic pain [36]. Consequently, the ω-CTX derivative ziconotide (Prialt, for intrathecal administration) was approved by the FDA in 2000 to treat severe and refractory chronic pain [37–39]. Unfortunately, this drug produces severe side effects, such as hallucinations or other psychiatric symptoms, that are associated with its principal MoA. Thus, it would be preferrable to induce an indirect partial reduction of Ca^2+^ influx via these widely expressed VDCCs in the CNS [36–39].

Therefore, we now extend our study of the effects of selective A_3_AR agonists on N-type Ca^2+^ channels in rat DRG neurons to a specialized agonist that has a “warhead” for covalent binding with the receptor, i.e., *N*^6^-(3-isothiocyanatobenzyl)-5′-*N*-methylcarboxamidoadenosine (ICBM). It was previously demonstrated that ICBM binds irreversibly and selectively to the rat A_3_AR in transfected Chinese hamster ovary (CHO) cell membranes and in membranes from rat basophilic cells that endogenously express the receptor [40].

## Materials and methods

### Cell culture

All animal experiments satisfied the regulatory requirements of the European Parliament (Directive 2010/63/EU), European Union Council (September 22, 2010) and Italian Animal Welfare Law (DL 26/2014). The protocol received approval from the Institutional Animal Care and Use Committee (Univ. Florence) and the Italian Ministry of Health. Animal suffering and the animal number required for reproducibility were minimized. Male and female Wistar rats (age 3–4 weeks) were obtained from Envigo, Udine, Italy, and housed under temperature- and humidity-control (12-h dark/light cycle) and allowed free food and water access) and euthanized by cervical dislocation. Primary DRG neurons were isolated and cultured as reported [32]. In brief, ganglia were excised bilaterally and treated with type 1A collagenase and trypsin (Sigma-Aldrich, Milan, Italy, 2 and 1 mg/ml in Hank’s balanced salt solution, respectively, followed by incubation at 37 °C for 25–35 min. Cells were centrifuged and the pellet resuspended and mechanically digested in Dulbecco’s Modified Eagle’s Medium (DMEM) containing 20% heat-inactivated FBS, penicillin (100 U/ml), streptomycin (0.1 mg/ml), and L-Gln (2 mM). The processed neurons were further centrifuged at 1200 g for 6 min and then re-suspended in supplemented DMEM, additionally containing mouse nerve growth factor (100 ng/ml) and cytosine-β-D-arabino-furanoside as the free base (2.5 mM). The neurons were plated on glass coverslips (13-mm) coated with poly-L-lysine (8.3 mM) and laminin (5 mM) and cultured for 1–2 days prior to immunohistochemical or electrophysiological experiments.

### Electrophysiology

Patch-clamp recording in whole cells was carried out according to a published procedure [32]. The following salts and buffer were present in the extracellular solution: CsCl (4 mM); NaCl (147 mM); CaCl_2_ (2 mM); MgCl_2_ (1 mM); D-glucose (10 mM); and HEPES (10 mM) and adjusted to pH 7.4 by using CsOH. The solution present in the pipette was CsCl (120 mM); Mg_2_-ATP (3 mM); EGTA (10 mM); and HEPES (10 mM; pH 7.4 with CsOH). To record I_Ca_, tetrodotoxin (TTX, 1 µM) was added to block TTX-sensitive sodium channels (Nav1.1, 1.2, 1.3, 1.4, 1.5, 1.6, 1.7), and A887826 (200 nM) was added to block TTX-insensitive (Nav 1.8, 1.9) channels. Furthermore, to isolate N-type Ca^2+^ channels, 100 mM Ni^2+^ in the form of NiCl_2_ was added to the extracellular solution, as it blocks T-type Ca^2+^ channels that are not involved in A_3_AR-mediated effects of ICBM and other A_3_AR agonists.

Neurons were introduced in a 1-ml platform-mounted recording chamber in an inverted microscope (Olympus CKX41, Milan, Italy), and superfusion was controlled using a 3-way perfusion valve (Harvard Apparatus, Holliston, MA, USA) to maintain a 2 ml/min flow rate. Electrodes (borosilicate glass, Harvard Apparatus) were formed to provide a to a final tip resistance of 2–4 MΩ using a P-87 Micropipette Puller (Sutter Instruments, Novato, CA, USA). Electrophysiological experiments were conducted at 21 ± 1 °C (ambient temperature). Signals were passed through an Axopatch 200B amplifier (Axon Instruments, Union City, CA, USA), with 10 kHz low-pass filtering, and the stored recordings were analyzed using pClamp 9.2 software purchased from Axon Instruments, Inc. (Union City, CA). Recording of fast hyperpolarizing voltage pulses (from − 60 to − 70 Mv, 40-ms duration) enabled analysis of series resistance (Rs), membrane resistance (Rm), and membrane capacitance (Cm). The analysis was limited to cells that displayed stable Cm and Rs levels at all experimental stages. VDCC currents were evoked with 0-mV step depolarizations (200 ms, Vh =  − 65 mV) every 30 s under Cs^+^-replacement conditions, an interval compatible with reproducible and stable I_Ca_ recording and with minimal Ca^2+^ current run down. The Ca^2+^ current-to-voltage relationship was captured by eliciting 10 depolarizing voltage steps (each 200 ms duration in 10 mV increments at 5 s intervals) from − 50 to + 50 Mv starting from − 65 mV (Vh).

Averaged currents expressed as pA/pF were obtained by normalizing cell capacitance and the neuronal diameter was also approximated from the cell capacitance by assuming a roughly spherical cell shape according to the calculated Cm for all biological membranes of 1 µF/cm^2^ and the equation for the total surface area of a sphere (A = 4 πr^2^).

### Immunocytochemical analysis

DRG neurons were cultured on 13-mm diameter coverslips and fixed (10 min at ambient temperature) with paraformaldehyde (4%) in 0.1 M PBS (Pan-Biotech, Milan, Italy). Cells were then PBS-washed twice and incubated in PBS solution containing 0.25% Triton X-100 (Merck Life Science S.r.l., Milano, Italy; PBST). After triply washing with PBS, the neurons were incubated with 10% goat serum (Merck Life Science S.r.l) in PBST (PBST-GS) for 30 min, to block nonspecific antibody binding, and then incubated at ambient temperature for 2.5 h in PBST-GS containing primary rabbit anti-A_3_R antibody (Alomone Labs, Jerusalem, Israel, diluted 1:100) plus mouse anti-β3-tubulin primary antibody (Cell Signaling Technology, Danvers, MA, USA, diluted 1:400). The cells were washed PBS (3X) and incubated (1 h at ambient temperature) with specific secondary antibodies: AlexaFluor488-labeled anti-mouse and AlexaFluor555-labeled anti-rabbit (AbCam, Cambridge, UK), each diluted 1:500 in PBST-GS. Coverslips were mounted with Fluoroshield (Merck Life Science S.r.l) containing the DNA stain DAPI to identify cell nuclei. Immunocytochemical images were captured using a TSC SP8 confocal microscope (Leica Microsystems, Mannheim, Germany), equipped with a × 63 oil-immersion objective (NA 1.40). The collected images were then analyzed using open-source software (ImageJ, version 1.49v, National Institutes of Health, Bethesda, MD, USA). Incubation of fixed cells in the presence of 2˚ antibodies and DAPI, alone, was used as a control to exclude nonspecific binding.

### Drugs

2-Chloro-*N*^6^-(3-iodobenzyl)-adenosine-5′-N-methyluronamide (Cl-IB-MECA; Merck Life Science S.r.l.) was used as a selective A_3_AR agonist. *N*^6^-(3-Isothiocyanatobenzyl)-5′-*N*-methylcarboxamidoadenosine (ICBM) was synthesized as reported [40] and was used as a selective A_3_AR agonist. Stock solutions of ICBM in DMSO were prepared and stored as small aliquots at − 20 °C and warmed to RT immediately before use, to avoid decomposition associated with freeze–thaw cycles [41]. The K_i_ values of this compound were described in rat cloned A_1_AR, A_2A_AR and A_3_AR stably transfected in CHO cells (K_i_ values are 145, 272, and 10.0 nM, respectively).

Tetrodotoxin (**TTX**; Tocris, Bio-Techne S.r.l., Bristol, UK) was used to block Na^+^ channels. 5-(4-butoxy-3-chlorophenyl)-N-[[2-(4-morpholinyl)-3-pyridinyl]methyl]-3-pyridinecarboxamide (A887826: Merck Life Science S.r.l.) was used to block TTX-insensitive Na^+^ channels (Nav1.5; Nav1.8; Nav1.9). 3-Propyl-6-ethyl-5-[(ethylthio)carbonyl]-2-phenyl-4-propyl-3-pyridinecarboxylate (MRS1523; Sigma-Aldrich, St. Louis, MO, USA) was used as a selective A_3_AR antagonist. 8-Cyclopentyl-1,3-dipropylxanthine (DPCPX; Merck Life Science S.r.l.) was used as a selective A_1_AR antagonist. DPCPX was added to all electrophysiological solutions to prevent A_1_AR activation [32].

### Statistical analysis

Shapiro–Wilk normality test was performed to check data distribution. As all data resulted normally distributed, statistical analysis was made uniformly with parametric tests. Data are expressed as mean ± SEM. Student paired or unpaired *t* tests and one-way analysis of variance (ANOVA) followed by Bonferroni analysis were performed, as appropriated, to determine statistical significance (set at *P* < 0.05). Data were analyzed using GraphPad Prism (GraphPad Software, San Diego, CA) software.

## Results

Present data were collected from 57 DRG neurons isolated from 12 rats. Averaged membrane resistance (Rm) was 984.17 MΩ and membrane capacitance (Cm) was 22.51 pF (*n* = 57).

**Fig. 1:**
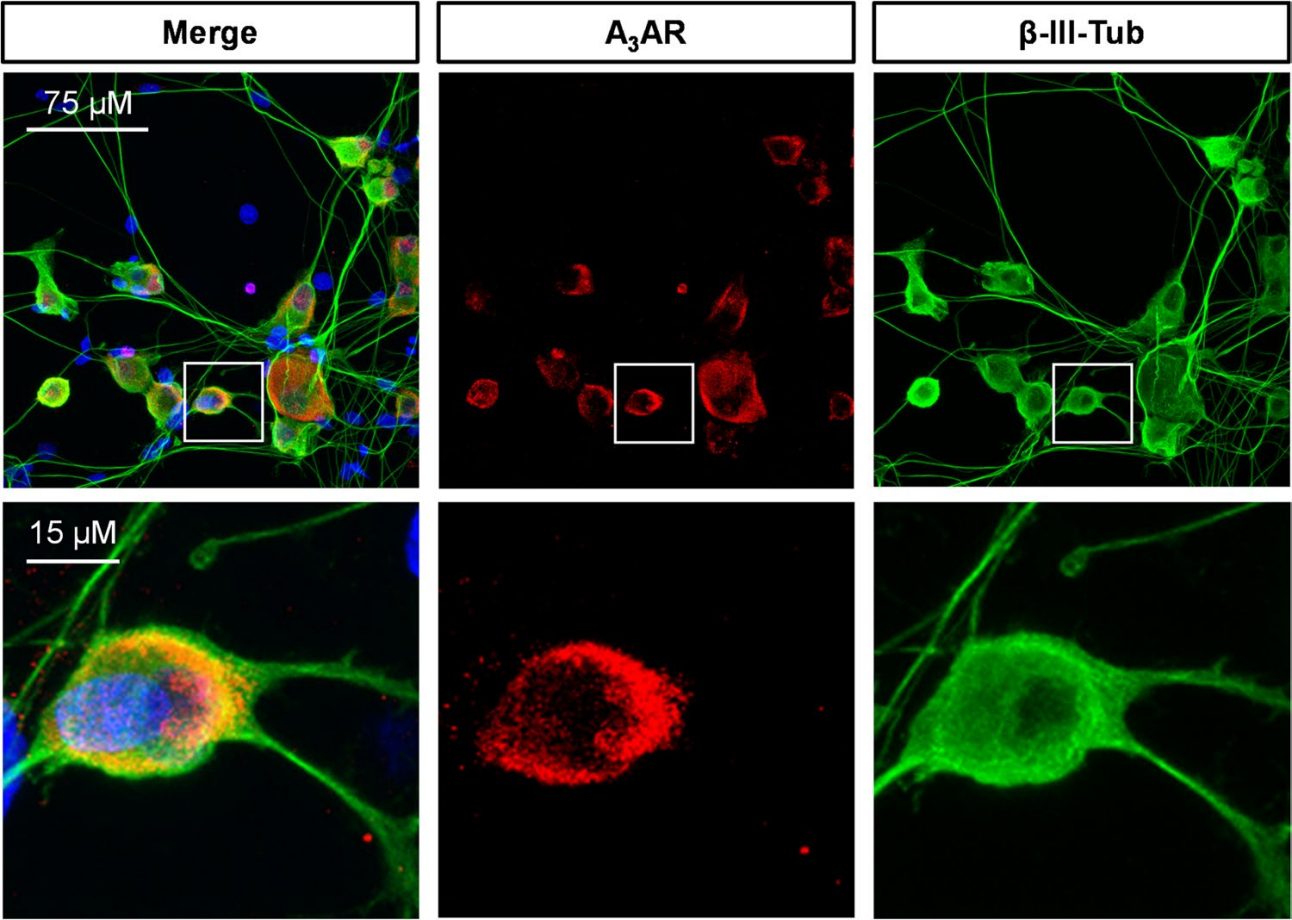
A_3_AR expression on cultured rat DRG neurons. Confocal imaging analysis showing 20X (upper panels) and respective magnification of A_3_AR-like immunofluorescence (red) on β-III-tubulin (green)-expressing DRG neuronal cultures. Cells nuclei are marked with DAPI (blue)

**Fig. 2:**
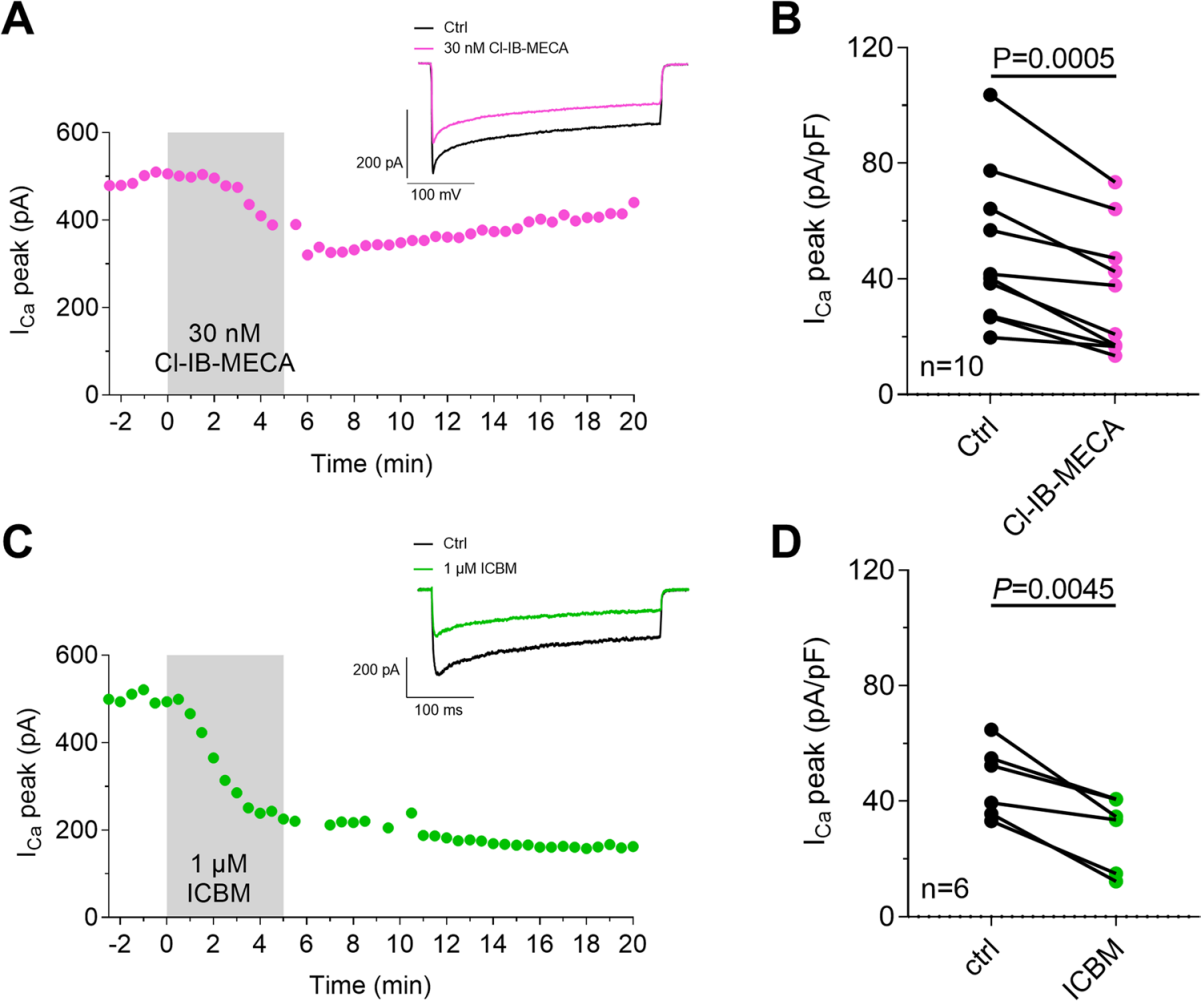
The new, irreversible, A_3_AR agonist ICBM inhibits N-type I_Ca_ in rat DRG neurons. **A**, **C** Time courses of peak Ca^2+^ current amplitude (I_Ca_ peak) evoked by a 0 mV step depolarization in a typical DRG neuron once every 30 s before, during, and after the application of the prototypical competitive A_3_AR agonist Cl-IB-MECA (100 nM, **A**) or the irreversible A_3_AR agonist ICBM (1 µM, **C**). Upper panels: original current traces recorded in respective cells before and during A_3_AR agonist application (Cl-IB-MECA, upper panel in **A**; ICBM, upper panel in **C**). Scale bars: 200 pA, 100 ms. **B**, **D** Pooled data (mean ± SEM) of peak I_Ca_ measured before (baseline, bsl) or during Cl-IB-MECA (30 nM, *n* = 10; **B**) or ICBM (1 µM, *n* = 6; **D**) application. *P* values are referred to paired Student’s *t*-test

First, we confirmed by confocal microscopy that isolated rat DRG neurons express the A_3_AR (Fig. 1) and that the prototypical A_3_AR agonist, Cl-IB-MECA, significantly decreases I_Ca_ (Fig. 2A–B) in these cells, as previously demonstrated by us [32]. Consistent with previous data, the Cl-IB-MECA effect peaked within 5 min of application and was partially reversed after 10 min of drug washout (Fig. 2A). In another set of experiments, we applied a newly synthesized batch of the highly selective and irreversibly binding A_3_AR agonist ICBM [40]. Similarly, to Cl-IB-MECA, ICBM significantly decreases peak I_Ca_, but, unlike the prototypical agonist, its effect was not reversible after ≤ 15 min washout (Fig. 2C–D).

**Fig. 3:**
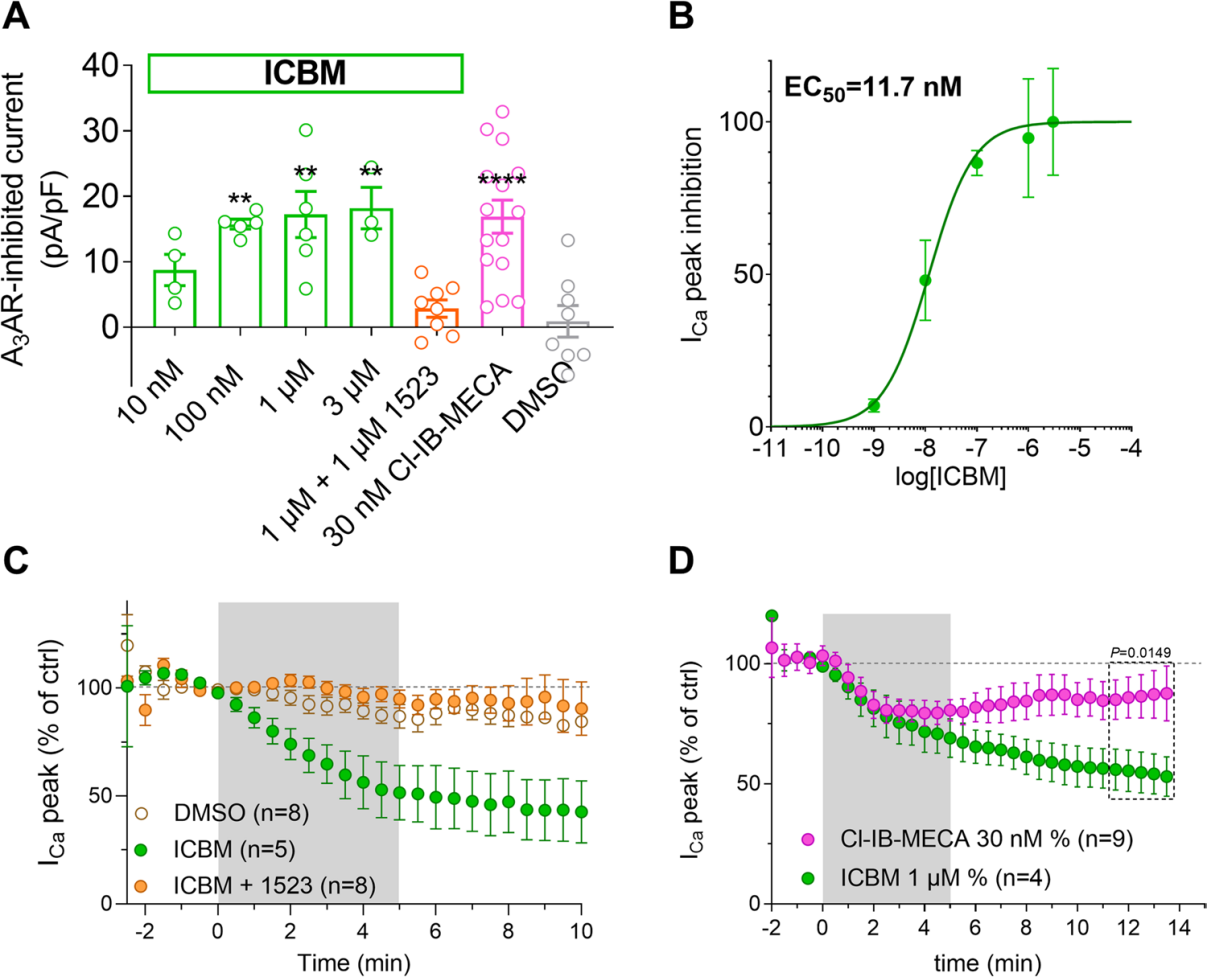
The effect of ICBM is concentration-dependent, sensitive to the A_3_AR antagonist MRS1523, and, differently from Cl-IB-MECA, not reversible after washout. **A** Pooled data (mean ± SEM) of I_Ca_ inhibition in DRG neurons superfused with different concentrations of the irreversible A_3_AR agonist ICBM (0.01–3 µM), by a maximal concentration (30 nM) of the prototypical A_3_AR agonist Cl-IB-MECA, by a maximal concentration (1 µM) of ICBM in the presence of the A_3_AR antagonist MRS1523 (1 µM) or by vehicle (0.1% DMSO). ** *P* < 0.01; **** *P* < 0.0001 vs DMSO, one-way ANOVA, Bonferroni post hoc test. **B** Concentration–response curve of the effect of ICBM on I_Ca_ inhibition. EC_50_ = 22.9 nM (confidence limit 4.1–33.5 nM). **C** Averaged time-courses of peak I_Ca_ amplitude (peak I_Ca_), expressed as % of baseline (bsl) values, measured before, during, or after the application A_3_AR ligands or their vehicle. **D** Effect of prolonged washout of Cl-IB-MECA (30 nM) or ICBM (1 µM) on averaged time-courses of peak I_Ca_ before, during, and after the application of Cl-IB-MECA (30 nM, purple circles, *n* = 10) or the new, irreversible, A_3_AR agonist ICBM (1 µM, green circles, *n* = 4). *P* value refers to an unpaired Student’s *t*-test

The inhibitory effect of ICBM (0.01–3 µM) on I_Ca_ was concentration-dependent (Fig. 3A), with an EC_50_ of 11.7 nM (confidence limit 4.1–33.5 nM; Fig. 3B) and was prevented by the selective A_3_AR antagonist MRS1523 (1 µM; Fig. 3A and C). As shown in Fig. 3D, the averaged ICBM-mediated I_Ca_ inhibition was, unlike the Cl-IB-MECA-mediated effect, not reversible after 9–11 min washout. Hence, functional data are consistent with previous binding experiments demonstrating irreversible displacement of radioligand binding by ICBM on A_3_AR-transfected CHO cells [40].

**Fig. 4:**
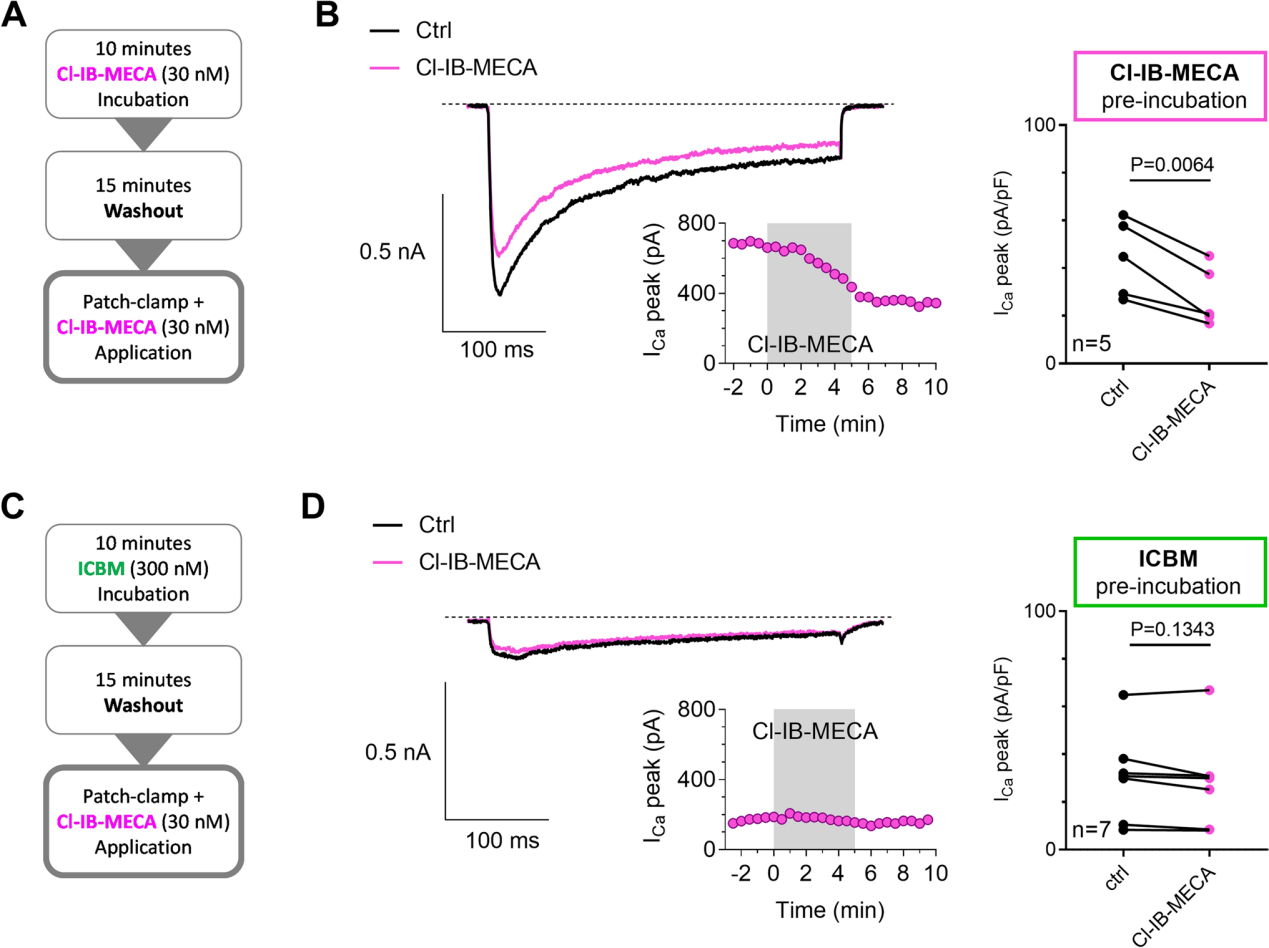
Pre-exposure of DRG neurons to the new A_3_AR agonist ICBM irreversibly blocks I_Ca_ and prevents the effect of a successive application of Cl-IB-MECA. **A**, **C** Experimental protocol used to compare the long-term effects of the prototypical A_3_AR agonist Cl-IB-MECA (30 nM) or the new, irreversible, A_3_AR agonist ICBM (1 µM) on I_Ca_ inhibition in DRG neurons. The protocol consisted of a 10-min exposure of DRG cultures to the A_3_AR agonist (added to the culture medium), followed by a 15-min washout (removal of the A_3_AR agonist-containing medium and perfusion of DRG neurons with the extracellular patch-clamp solution in the recording chamber) during which a stable baseline of peak I_Ca_ amplitude (peak I_Ca_) was acquired by whole-cell patch-clamp recordings. Hence, the effect of a subsequent exposure to the prototypical A_3_AR agonist Cl-IB-MECA (30 nM) on I_Ca_ was assessed in the same cell. **B**, **D** Original current traces recorded in a Cl-IB-MECA (**B**) or in a ICBM (**D**) pre-incubated DRG neuron before and during the Cl-IB-MECA (30 nM) application. Scale bars: 500 pA; 100 ms. Lower panels: typical time course of I_Ca_ peak evoked in respective cells. Right panels: pooled data (mean ± SEM) of I_Ca_ peak measured before (baseline, bsl) or during the Cl-IB-MECA (30 nM, *n* = 5; B) or ICBM (1 µM, *n* = 7; **D**) application. *P* value refers to paired Student’s *t*-test

To corroborate the hypothesis of irreversible A_3_AR-mediated I_Ca_ inhibition after acute exposure to ICBM, we added efficacious concentrations of Cl-IB-MECA (30 nM) or ICBM (1 µM) to the culture medium of DRG neurons for 10 min, then the A_3_AR agonist was removed by replacement with control medium for 15 min. Then, cells were transferred to a recording chamber for whole-cell patch recordings and the effect of a subsequent 7 min Cl-IB-MECA application on I_Ca_ was evaluated (Fig. 4A). As shown in Fig. 4B, the application of 30 nM Cl-IB-MECA after a previous incubation with Cl-IB-MECA (Cl-IB-MECA pre-incubation 10 min, 30 nM) was still able to decrease I_Ca_ in DRG neurons (*P* = 0.0064; Fig. 4B). In contrast, no effect of Cl-IB-MECA was observed in ICBM-pre-incubated (10 µM; 10 min) cells (*P* = 0.1343; Fig. 4C, D).

**Fig. 5:**
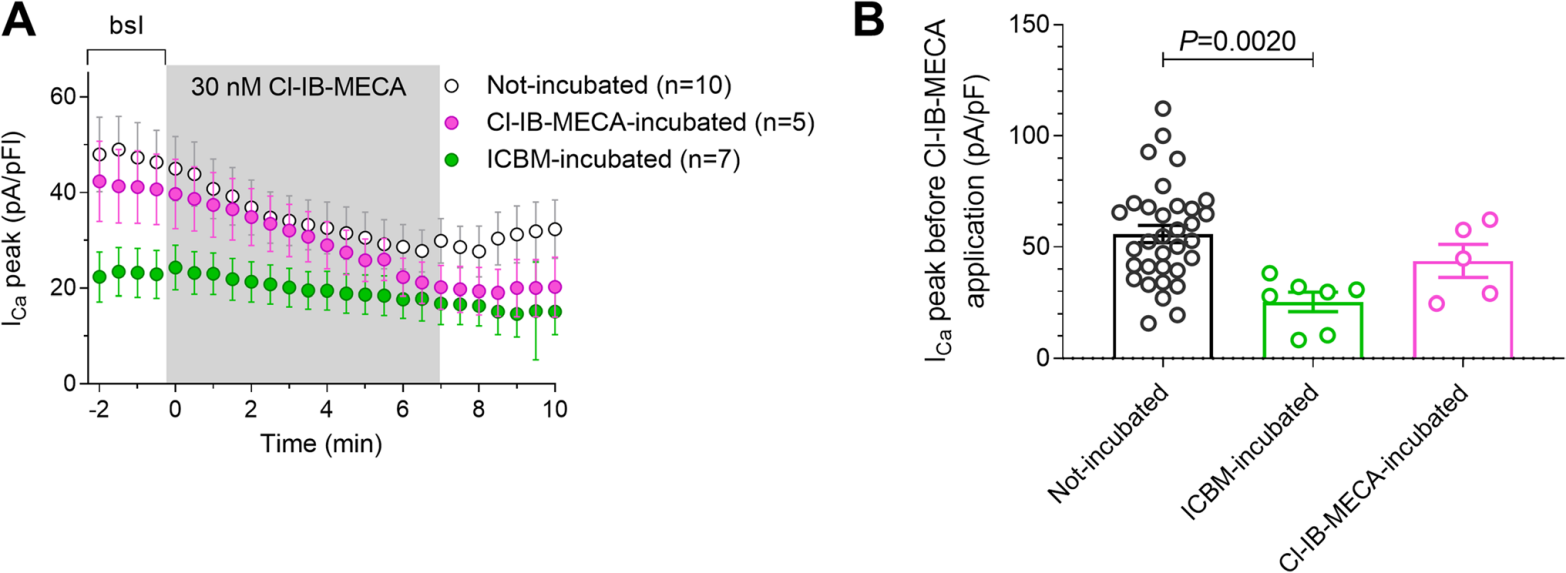
Functional irreversibility of ICBM effect on I_Ca_ in DRG neurons. **A** Averaged (mean ± SEM) time-courses of I_Ca_ peak amplitude (I_Ca_ peak) recorded before, during, and after application of Cl-IB-MECA (30 nM) in Cl-IB-MECA pre-incubated (30 nM, 10 min, purple circles, *n* = 5) cells, in ICBM pre-incubated (1 µM, 10 min, green circles, *n* = 4) cells, or in not-incubated (empty circles, *n* = 10) cells. **B** Pooled data of I_Ca_ peak measured during baseline (bsl; i.e., before the Cl-IB-MECA application) in respective experimental groups. *P* value refers to one-way ANOVA, Bonferroni post hoc test vs not-incubated cells

It is also worth to note that the peak I_Ca_ amplitude evoked in ICBM-pre-incubated DRG neurons during baseline (bsl; i.e., before the 7 min Cl-IB-MECA application; Fig. 5A) was significantly smaller than that measured in non-incubated cells (Fig. 5B, *P* = 0.0073, One-way ANOVA, Bonferroni post-test). These results indicate that, after a 10 min ICBM pre-incubation, I_Ca_ inhibition by A_3_AR activation persists, thus occluding the effect of a subsequent Cl-IB-MECA treatment.

## Discussion

In the present work, we investigated the functional effect of ICBM, a new A_3_AR agonist designed for covalent binding to the receptor, on DRG neurons by means of electrophysiological recordings. We demonstrated that ICBM inhibits I_Ca_ evoked by membrane depolarization in isolated rat DRG neurons, an effect similar to that observed in the presence of the non-covalent prototypical A_3_AR agonist, Cl-IB-MECA [32]. However, unlike Cl-IB-MECA, ICBM-mediated inhibition of I_Ca_ persisted after drug removal, indicating a long-lasting effect of this compound.

The A_3_AR is expressed in diverse tissues at relatively low levels, compared to other adenosine receptors. Genomic analysis of the expression of the A_3_AR gene in various human tissues shows highest levels in testes, the spinal cord, and various brain regions, bladder, lung, adipose tissue, and whole blood [42, 43]. Here, we also confirmed by histochemical analysis that rat DRG neurons express the A_3_AR (Fig. 1). Moreover, the A_3_AR structure displays notable interspecies variations in ligand recognition [44]. For all these reasons, as well as the diverse pharmacological profiles of the species homologs, it has been challenging to characterize A_3_AR ligands.

In 1994, Ji et al. synthesized a new A_3_AR agonist (ICBM) containing a chemically reactive isothiocyanate group that was found to bind this adenosine receptor subtype selectively and irreversibly [40]. Isothiocyanate groups on A_1_AR and A_2A_AR ligands were previously shown to induce irreversible receptor binding. ICBM showed a high affinity for rat A_3_AR in membranes of transfected CHO cells (10.0 ± 2.3 nM) and RBL basophilic cells. Furthermore, preincubation of transfected CHO cell membranes induced a concentration-dependent, irreversible antagonism that was demonstrated by the failure of repeated washing to regenerate the A_3_AR binding sites. Moreover, a preincubation with 100 nM ICBM followed by washing resulted in diminished B_max_, representing a loss in the density of binding sites of 41% [40]. We have extended the earlier findings of irreversible binding of ICBM in rat A_3_AR-expressing cell membranes to introduce a promising potential therapeutic direction for a covalent A_3_AR agonists, i.e., in pain therapy.

Here, we examined the functional effects of this isothiocyanate derivative on voltage-dependent I_Ca_ in DRG neurons. In line with previous data, ICBM mimicked the effect of Cl-IB-MECA in inhibiting I_Ca_ in these cells. Furthermore, the irreversible binding of ICBM to the A_3_AR was demonstrated as follows: (i) when cells were pre-incubated with ICBM, washed for 10 min, and then challenged with acute Cl-IB-MECA exposure, Cl-IB-MECA was ineffective in inhibiting I_Ca_; (ii) the amplitude of I_Ca_ in DRG neurons preincubated with ICBM was significantly smaller than untreated and Cl-IB-MECA-preincubated cells. Thus, we confirmed, at a functional level, that the covalent binding of ICBM to the A_3_AR produces an irreversible inhibition of I_Ca_.

It is well recognized that the A_3_AR undergoes rapid agonist-induced desensitization and internalization in model cell systems [45]. Following these events, the receptor undergoes recycling with re-sensitization of receptor responsiveness, while after prolonged (≤ 24 h) agonist exposure the receptor undergoes downregulation [45]. However, activation of many GPCRs, including A_3_AR, can lead to β-arrestin binding and sequestration of the receptor from the cell surface, but some receptors can still signal to the cAMP pathway even after receptor internalization [46], as found for other receptor subtypes [47–49]. Stoddart and co-authors demonstrated that the highly conserved tryptophan (W6.48) in TM6 is essential for the active conformation of A_3_AR to interact with β-arrestin2, and necessary for it to undergo receptor internalization [46]. In addition, their data showed that individual agonists elicit different changes in the position of this residue, with consequent implications for their ability to activate G_i_-coupling and receptor internalization [46]. Nevertheless, the prolonged activation of the A_3_AR by ICBM is striking, and further studies will be informative about the cellular location and signaling properties of the covalently ligated receptor. Future studies could also probe the site of covalent reactivity of the isothiocyanate group on the receptor protein. Also, other electrophilic groups might be suitable for incorporation in covalent A_3_AR agonists in addition to an isothiocyanate.

From a pre-clinical perspective, it is recognized that A_1_AR and A_3_AR agonists inhibit I_Ca_ in DRG neurons and play an important anti-algetic role in several pain models [5, 32, 50, 51]. It should be noted that A_3_AR activation is known to selectively inhibit N-type Ca^2+^ channels, as the effect of the prototypical A_3_AR agonist Cl-IB-MECA is prevented by the ω-CTX analogue PD173212 but not by the L-type blocker lacidipine [32]. Hence, A_3_AR stimulation on DRG neurons would selectively decrease N-type Ca^2+^ currents and, in turn, neurotransmitter/neuropeptide release at the synapse, since it was demonstrated that the block of these currents prevents sensory neurons from releasing of the pain-related neuropeptides, such as substance P and calcitonin gene-related peptide [52, 53]. Ziconotide, the preferred ω-CTX analogue, is currently being used clinically in the USA as an intrathecal pain reliever for chronic pain [37, 54]. It should be noted that a direct inhibition of N-type Ca^2+^ channels, such as that accomplished by ziconotide or ω-CTXs, is related to adverse side effects (psychological and neuropsychiatric symptoms including depression, cognitive impairment, and hallucinations; anxiety; panic attacks; ataxia; asthenia; headache; and dysesthesia) [55]. Interestingly, an “indirect” VDCC modulation, as that accomplished by A_3_AR activation, could represent a suitable approach to pain control with milder adverse effects. In addition, the A_1_AR has shown cardiovascular side effects in clinical trials [14]. On the other hand, unlike A_1_AR, activation of the A_3_AR in humans by potent, selective, and orally accessible A_3_AR agonists is not linked to cardiac or hemodynamic adverse effects [14], therefore representing a promising treatment for chronic pain of different etiologies. Moreover, A_3_AR agonists proved relatively free from adverse effects in phase II/III clinical trials for other pathologies [14, 56] and are considered to be encouraging therapeutic candidates in advanced phases of clinical research, possibly due to the lower expression of A_3_AR in peripheral tissue [57]. It has been demonstrated in in vivo models of neuropathic or chronic pain that A_3_AR stimulation has profound anti-hyperalgesic effects, by central and/or peripheral mechanisms of action [3, 5, 19, 20, 30, 32, 58–60]. Based on the present data, ICBM might be an interesting compound to be investigated as a non-narcotic pain reliever, because by irreversibly binding to the A_3_AR, it produces long-lasting A_3_AR activation able to provide pain control over a longer time span after drug administration.

From a translational perspective, irreversibly binding A_3_AR agonists such as ICBM may represent an innovative, beneficial strategy to achieve efficacious chronic pain control as A_3_AR agonists, i.e., Cl-IB-MECA and IB-MECA, already proved safe and secure in clinical trials for other pathologies. Irreversibly binding drugs containing a covalent warhead are now coming into focus in the pharmaceutical industry as a viable drug discovery approach [61]. ICBM could be viewed as a prototypical covalent A_3_AR agonist, on which future molecules can be designed. Of note, possible limitations of this drug may consist, for example, of a longer-lasting effect which is difficult to manage in case of over dosage. Furthermore, a different adverse effect profile might be hypothesized in comparison to prototypical (reversible) A_3_AR agonists such as IB-MECA and Cl-IB-MECA. Of note, the fact that this compound irreversibly binds to the A_3_AR might be advantageous to defer the time or decrease the dosage of drug administration, especially in the case of chronic diseases, such as neuropathic pain. Hence, more pre-clinical studies are needed to define the functional effect/s of ICBM in animal models.

## Data Availability

The data that support the findings of this study are available from the corresponding author upon reasonable request. Besides, all data discussed in this article are available in cited publications.

## Abbreviations

AR: Adenosine receptor
CHO: Chinese hamster ovary
Cl-IB-MECA: 2-Chloro-*N*^6^-(3-iodobenzyl)adenosine-5′-*N*-methylcarboxamide
Cm: Membrane capacitance
ω-CTX: ω-Conotoxin
DAPI: 4′,6-Diamidino-2-phenylindole
DPCPX: 8-Cyclopentyl-1,3-dipropylxanthine
DRG: Dorsal root ganglion
GPCR: G protein-coupled receptor
I_Ca_: Ca^2+^ currents
ICBM: *N*^6^-(3-Isothiocyanatobenzyl)-5′-*N*-methylcarboxamidoadenosine
MRS1523: 3-Propyl-6-ethyl-5-[(ethylthio)carbonyl]-2-phenyl-4-propyl-3-pyridinecarboxylate
Rm: Membrane resistance
Rs: Series resistance
TTX: Tetrodotoxin
VDCC: Voltage-dependent Ca^2+^ channel

## References

[1] Goldberg DS, McGee SJ (2011). Pain as a global public health priority. BMC Public Health.

[2] di Cesare ML, Pacini A, Corti F (2015). Antineuropathic profile of N-palmitoylethanolamine in a rat model of oxaliplatin-induced neurotoxicity. PLoS One.

[3] Paoletta S, Tosh DK, Finley A (2013). Rational design of sulfonated A3 adenosine receptor-selective nucleosides as pharmacological tools to study chronic neuropathic pain. J Med Chem.

[4] Fredholm BB, IJzerman AP, Jacobson KA (2011). International union of basic and clinical pharmacology. LXXXI. Nomenclature and Classification of Adenosine Receptors–An Update. Pharmacol Rev.

[5] Coppi E, Cherchi F, Lucarini E (2021). Uncovering the mechanisms of adenosine receptor-mediated pain control: focus on the A3 receptor subtype. Int J Mol Sci.

[6] Coppi E, Cherchi F, Venturini M (2022). Therapeutic potential of highly selective A3 adenosine receptor ligands in the central and peripheral nervous system. Molecules.

[7] Dickenson A, Suzuki R, Reeve A (2000). Adenosine as a potential analgesic target in inflammatory and neuropathic pains. CNS Drugs.

[8] Luongo L, Salvemini D (2018). Targeting metabotropic adenosine receptors for neuropathic pain: focus on A2A. Brain Behav Immun.

[9] Abo-Salem OM, Hayallah AM, Bilkei-Gorzo A (2004). Antinociceptive effects of novel A2B adenosine receptor antagonists. J Pharmacol Exp Ther.

[10] Savegnago L, Jesse CR, Nogueira CW (2008). Caffeine and a selective adenosine A2B receptor antagonist but not imidazoline receptor antagonists modulate antinociception induced by diphenyl diselenide in mice. Neurosci Lett.

[11] Deb PK, Deka S, Borah P (2019). Medicinal chemistry and therapeutic potential of agonists, antagonists and allosteric modulators of A1 adenosine receptor: current status and perspectives. Curr Pharm Des.

[12] Fishman P, Bar-Yehuda S, Liang BT, Jacobson KA (2012). Pharmacological and therapeutic effects of A3 adenosine receptor agonists. Drug Discov Today.

[13] Jacobson KA, Gao ZG (2006). Adenosine receptors as therapeutic targets. Nat Rev Drug Discov.

[14] Jacobson KA, Tosh DK, Jain S, Gao ZG (2019). Historical and current adenosine receptor agonists in preclinical and clinical development. Front Cell Neurosci.

[15] Silverman MH, Strand V, Markovits D (2008). Clinical evidence for utilization of the A3 adenosine receptor as a target to treat rheumatoid arthritis: data from a phase II clinical trial. J Rheumatol.

[16] Kleindorfer D, Lindsell CJ, Brass L (2008). National US estimates of recombinant tissue plasminogen activator use: ICD-9 codes substantially underestimate. Stroke.

[17] Yenari MA, Han HS (2012). Neuroprotective mechanisms of hypothermia in brain ischaemia. Nat Rev Neurosci.

[18] Diller KR, Zhu L (2009) Hypothermia therapy for brain injury. 11:135–162.10.1146/ANNUREV-BIOENG-061008-124908 10.1146/annurev-bioeng-061008-124908 19400711

[19] Lucarini E, Coppi E, Micheli L (2020). Acute visceral pain relief mediated by A3AR agonists in rats: involvement of N-type voltage-gated calcium channels. Pain.

[20] Durante M, Squillace S, Lauro F (2021). Adenosine A3 agonists reverse neuropathic pain via T cell–mediated production of IL-10. J Clin Invest.

[21] Müller CE, Jacobson KA (2011). Recent developments in adenosine receptor ligands and their potential as novel drugs. Biochim et Biophys Acta (BBA) - Biomembranes.

[22] Jacobson KA, Klutz AM, Tosh DK (2009). Medicinal chemistry of the A3 adenosine receptor: agonists, antagonists, and receptor engineering. Handb Exp Pharmacol.

[23] Kim HO, Xiao-duo J, Siddiqi SM (1994). 2-Substitution of N6-benzyladenosine-5′-uronamides enhances selectivity for A3 adenosine receptors. J Med Chem.

[24] Jacobson KA, Ji XD, Li AH (2000). Methanocarba analogues of purine nucleosides as potent and selective adenosine receptor agonists. J Med Chem.

[25] Volpini R, Buccioni M, Dal Ben D (2009). Synthesis and biological evaluation of 2-alkynyl-N6-methyl- 5′-N-methylcarboxamidoadenosine derivatives as potent and highly selective agonists for the human adenosine A3 receptor. J Med Chem.

[26] Tosh DK, Finley A, Paoletta S (2014). In vivo phenotypic screening for treating chronic neuropathic pain: modification of C2-arylethynyl group of conformationally constrained A3 adenosine receptor agonists. J Med Chem.

[27] Tosh DK, Deflorian F, Phan K (2012). Structure-guided design of A3 adenosine receptor-selective nucleosides: combination of 2-arylethynyl and bicyclo[3.1.0]hexane substitutions. J Med Chem.

[28] Tosh DK, Paoletta S, Phan K (2012). Truncated nucleosides as A3 adenosine receptor ligands: combined 2-arylethynyl and bicyclohexane substitutions. ACS Med Chem Lett.

[29] Fedorova IM, Jacobson MA, Basile A, Jacobson KA (2003). Behavioral characterization of mice lacking the A3 adenosine receptor: sensitivity to hypoxic neurodegeneration. Cell Mol Neurobiol.

[30] Janes K, Esposito E, Doyle T (2014). A3 adenosine receptor agonist prevents the development of paclitaxel-induced neuropathic pain by modulating spinal glial-restricted redox-dependent signaling pathways. Pain.

[31] Ford A, Castonguay A, Cottet M (2015). Engagement of the GABA to KCC2 signaling pathway contributes to the analgesic effects of A3AR agonists in neuropathic pain. J Neurosci.

[32] Coppi E, Cherchi F, Fusco I (2019). Adenosine A3 receptor activation inhibits pronociceptive N-type Ca2+ currents and cell excitability in dorsal root ganglion neurons. Pain.

[33] Field MJ, Li Z, Schwarz JB (2007). Ca2+ channel α2-δ ligands for the treatment of neuropathic pain. J Med Chem.

[34] Vink S, Alewood PF (2012). No TitleTargeting voltage-gated calcium channels: developments in peptide and small-molecule inhibitors for the treatment of neuropathic pain. Br J Pharmacol.

[35] Heinke B, Balzer E, Sandkühler J (2004). Pre- and postsynaptic contributions of voltage-dependent Ca2+ channels to nociceptive transmission in rat spinal lamina I neurons. Eur J Neurosci.

[36] Hannon HE, Atchison WD (2013). Omega-conotoxins as experimental tools and therapeutics in pain management. Mar Drugs.

[37] Brookes ME, Eldabe S, Batterham A (2016). Ziconotide monotherapy: a systematic review of randomised controlled trials. Curr Neuropharmacol.

[38] Jain KK (2000). An evaluation of intrathecal ziconotide for the treatment of chronic pain. Expert Opin Investig Drugs.

[39] McDowell GC, Pope JE (2016). Intrathecal ziconotide: dosing and administration strategies in patients with refractory chronic pain. Neuromodulation.

[40] Ji XD, Gallorodriguez C, Jacobson KA (1994). A selective agonist affinity label for A3 adenosine receptors. Biochem Biophys Res Commun.

[41] Coppi E, Pedata F, Gibb AJ (2012) P2Y1 receptor modulation of Ca2+-activated K+ currents in medium-sized neurons from neonatal rat striatal slices. J Neurophysiol 107(3):1009-21. 10.1152/jn.00816.2009 10.1152/jn.00816.2009 PMC3289470 22131374

[42] Jacobson KA, Merighi S, Varani K (2018). A3 adenosine receptors as modulators of inflammation: from medicinal chemistry to therapy. Med Res Rev.

[43] Dixon AK, Gubitz AK, Sirinathsinghji DJS (1996). Tissue distribution of adenosine receptor mRNAs in the rat. Br J Pharmacol.

[44] Alnouri MW, Jepards S, Casari A (2015). Selectivity is species-dependent: characterization of standard agonists and antagonists at human, rat, and mouse adenosine receptors. Purinergic Signal.

[45] Trincavelli ML, Tuscano D, Marroni M (2002). A3 adenosine receptors in human astrocytoma cells: agonist-mediated desensitization, internalization, and down-regulation. Mol Pharmacol.

[46] Stoddart LA, Kellam B, Briddon SJ, Hill SJ (2014). Effect of a toggle switch mutation in TM6 of the human adenosine A3 receptor on Gi protein-dependent signalling and Gi-independent receptor internalization. Br J Pharmacol.

[47] Werthmann RC, Volpe S, Lohse MJ, Calebiro D (2012). Persistent cAMP signaling by internalized TSH receptors occurs in thyroid but not in HEK293 cells. FASEB J.

[48] Boutin A, Allen MD, Neumann S, Gershengorn MC (2012). Persistent signaling by thyrotropin-releasing hormone receptors correlates with G-protein and receptor levels. FASEB J.

[49] Mullershausen F, Zecri F, Cetin C (2009). Persistent signaling induced by FTY720-phosphate is mediated by internalized S1P1 receptors. Nat Chem Biol.

[50] Dolphin AC, Forda SR, Scott RH (1986). Calcium-dependent currents in cultured rat dorsal root ganglion neurones are inhibited by an adenosine analogue. J Physiol.

[51] MacDonald RL, Skerritt JH, Werz MA (1986). Adenosine agonists reduce voltage-dependent calcium conductance of mouse sensory neurones in cell culture. J Physiol.

[52] Evans AR, Nicol GD, Vasko MR (1996). Differential regulation of evoked peptide release by voltage-sensitive calcium channels in rat sensory neurons. Brain Res.

[53] Maggi CA, Giuliani S, Santicioli IP (1990). Effect of omega conotoxin on reflex responses mediated by activation of capsaicin-sensitive nerves of the rat urinary bladder and peptide release from the rat spinal cord. Neuroscience.

[54] Adler JA, Lotz NM (2017). Intrathecal pain management: a team-based approach. J Pain Res.

[55] Lynch SS, Cheng CM, Yee JL (2006). Formulary forum: intrathecal ziconotide for refractory chronic pain. Ann Pharmacother.

[56] Fishman P (2022). Drugs targeting the A3 adenosine receptor: human clinical study data. Molecules.

[57] Borea PA, Varani K, Vincenzi F (2015). The a3 adenosine receptor: history and perspectives. Pharmacol Rev.

[58] Little JW, Ford A, Symons-Liguori AM (2015). Endogenous adenosine A3 receptor activation selectively alleviates persistent pain states. Brain.

[59] Maloy C, Janes K, Bryant L (2014). (336) A3 adenosine receptor agonists reverse established oxaliplatin-induced neuropathic pain through an IL-10 mediated mechanism of action in spinal cord. J Pain.

[60] Tosh DK, Padia J, Salvemini D, Jacobson KA (2015). Efficient, large-scale synthesis and preclinical studies of MRS5698, a highly selective A3 adenosine receptor agonist that protects against chronic neuropathic pain. Purinergic Signal.

[61] Sutanto F, Konstantinidou M, Dömling A (2020). Covalent inhibitors: a rational approach to drug discovery. RSC Med Chem.

